# Supplementary material for: Development of a Multiple Temperature Sensors Device for the Characterization, Control and Monitoring of Microbiological Incubators
Source: Sensors (Basel). 2024 Nov 30;24(23):7671. doi: 10.3390/s24237671 (PMC11644915; doi:10.3390/s24237671)
Supplement: Supplementary file 1 [file sensors-24-07671-s001.zip › Supplementary_material.pdf]

Article

Development of a multiple temperature sensors device for the characterization, control and monitoring of microbiological incubators

Carolina Salinas Domján[1,2,*], Mauro A. Valente[1,2,3] and Marcelo R. Romero[2,4,5]

[1] Instituto de Física Enrique Gaviola (IFEG), CONICET-UNC, Córdoba X5000HUA, Argentina
[2] Laboratorio de Investigación e Instrumentación en Física Aplicada a la Medicina e Imágenes de Rayos X, LIIFAMIRx, FaMAF-UNC, Córdoba X5000HUA, Argentina
[3] Centro de Excelencia en Física e Ingeniería en Salud & Departamento de Ciencias Físicas, Universidad de la Frontera, Temuco Casilla 54-D 4811230, Chile
[4] Facultad de Ciencias Químicas, UNC, Córdoba X5000HUA, Argentina
[5] Instituto de Investigación y Desarrollo en Ingeniería de Procesos y Química Aplicada, IPQA – CONICET, Córdoba X5016GCA, Argentina

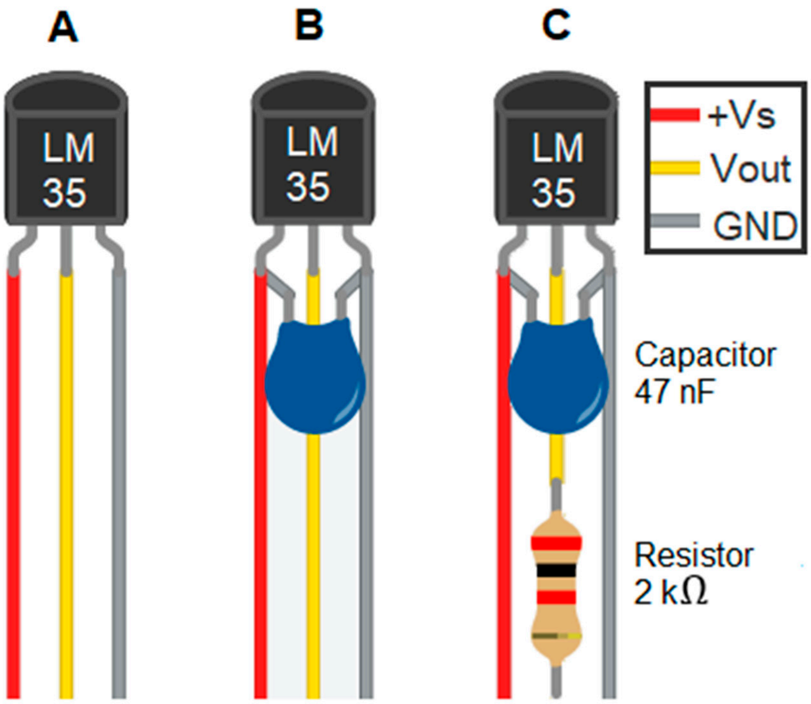

Figure S1: Proposed configurations for the LM35 sensor.

Table S1: Price list of components for the construction of devices with four temperature sensors.

Component	Unitary price (USD)	Total Price for device elaboration (USD)
LM35 sensor	1,55	6,20
2 kΩ Resistor	0,60	2,40
47 nF Capacitor	0,70	2,80
Audiopipe HWY 836 cable (10 f)	5,40	5,40
ARDUINO UNO board	8,99	8,99

Price control date: November 14, 2024

Link of price control: https://www.amazon.com/
