# Supplementary figures and images for: Development of a Multiple Temperature Sensors Device for the Characterization, Control and Monitoring of Microbiological Incubators
Source: Sensors (Basel). 2024 Nov 30;24(23):7671. doi: 10.3390/s24237671 (PMC11644915; doi:10.3390/s24237671)

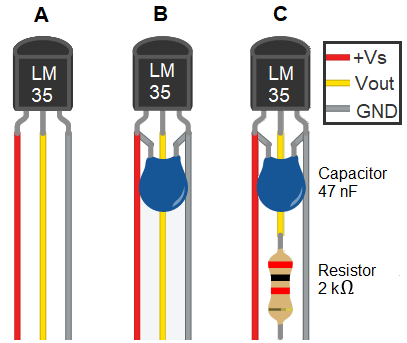

Supplement: Supplementary file 1 [file sensors-24-07671-s001.zip › FigureS1.png]
